# Building up a genomic surveillance platform for SARS-CoV-2 in the middle of a pandemic: a true North–South collaboration

**DOI:** 10.1136/bmjgh-2023-012589

**Published:** 2023-11-19

**Authors:** Waqasuddin Khan, Furqan Kabir, Samiah Kanwar, Fatima Aziz, Sahrish Muneer, Adil Kalam, Mehdia Nadeem Rajab Ali, Nadia Ansari, Manu Vanaerschot, Vida Ahyong, Liz Fahsbender, Katrina Kalantar, Allison Black, Abigail Glascock, Juliana Gil, Patrick Ayscue, Cristina Tato, Fyezah Jehan, Imran Nisar

**Affiliations:** 1 Pediatrics and Child Health, The Aga Khan University, Karachi, Sindh, Pakistan; 2 Biorepository and Omics Research Group, Medical College Pakistan, The Aga Khan University, Karachi, Sindh, Pakistan; 3 Chan Zuckerberg Biohub, San Francisco, California, USA; 4 The Chan Zuckerberg Initiative, Palo Alto, California, USA

**Keywords:** COVID-19, Health services research, Public Health, SARS, Epidemiology

## Abstract

Next-generation sequencing technology has revolutionised pathogen surveillance over the last two decades. However, the benefits are not equitably distributed, with developing countries lagging far behind in acquiring the required technology and analytical capacity. Recent declines in the cost associated with sequencing—equipment and running consumables have created an opportunity for broader adoption. During the COVID-19 pandemic, rapid diagnostics development and DNA sequencing revolutionised the ability to diagnose and sequence SARS-CoV-2 rapidly. Socioeconomic inequalities substantially impact the ability to sequence SARS-CoV-2 strains and undermine a developing country’s pandemic preparedness. Low- and middle-income countries face additional challenges in establishing, maintaining and expanding genomic surveillance. We present our experience of establishing a genomic surveillance system at the Aga Khan University, Karachi, Pakistan. Despite being at a leading health sciences research institute in the country, we encountered significant challenges. These were related to collecting standardised contextual data for SARS-CoV-2 samples, procuring sequencing reagents and consumables, and challenges with library preparation, sequencing and submission of high-quality SARS-CoV-2 genomes. Several technical roadblocks ensued during the implementation of the genomic surveillance framework, which were resolved in collaboration with our partners. High-quality genome sequences were then deposited on open-access platforms per the best practices. Subsequently, these efforts culminated in deploying Pakistan’s first SARS-CoV-2 phyllo surveillance map as a Nextstrain build. Our experience offers lessons for the successful development of Genomic Surveillance Infrastructure in resource-limited settings struck by a pandemic.

**Summary box**

- The use of Next-Generation Sequencing is essential for surveillance of the emergence and spread of SARS-CoV-2 variants.
- For informed national responses now and in the future, expanding sequencing capacity in resource-limited settings is required.
- Here we present our experiences, challenges and remedial strategies in building genomic surveillance, sequencing capacity and bioinformatics analysis in an academic setting.
- Positive COVID-19 samples were collected along with the metadata and sequenced, and bioinformatics analysis was performed afterward.
- High-quality genome and related metadata were submitted to Global Initiative on Sharing All Influenza Data, National Center for Biotechnology Information (NCBI) GenBank and the (European Nucleotide Archive at the European Bioinformatics Institute (ENA-EBI) COVID-19 portal.
- Challenges regarding procurement, deployment of the analysis pipeline and submission were encountered and resolved.
- The importance of genomics in controlling a disease outbreak has been learnt from the ongoing global pandemic.
- Our experience can be referenced in other low- and middle-income countries and for future pandemic preparedness.

## Introduction

Next-Generation Sequencing (NGS) is a powerful technique that can enable the rapid high-throughput sequencing of the SARS-CoV-2 RNA genome. Expanding SARS-CoV-2 genomic surveillance data along with clinically relevant contextual data will yield insights on the transmission patterns and pathophysiology of COVID-19.^1^ A cluster of unusual pneumonia cases detected in Wuhan, China, was first reported to the WHO in late December 2019. Due to the rapid and collaborative work of the scientific community and advancement in the field of sequencing, within 10 days after notification, the first SARS-CoV-2 genome sequence (Wuhan-Hu-1 strain) was available at the Global Initiative on Sharing All Influenza Data (GISAID).^2–5^ Moreover, Nextstrain uses the Wuhan-Hu-1 strain as a reference for building SARS-CoV-2 phylogenetic trees. The timely release of the first genomic sequences allowed the development of diagnostic protocols and the identification of the aetiological agent causing this outbreak.

Genomic surveillance of the evolving SARS-CoV-2 strains is vital for helping control the pandemic.^6^ The availability of sequenced genomes on an open-access platform, such as GISAID, is essential for efficient analysis of viral evolution and spread. The research community consistently accumulates sequences and analyses similarities and differences among these sequences. Collectively, this information is used to understand better how variants might impact public health. Wider geographical coverage allows detection of mutations that might affect virulence, pathogenesis, host range or immune escape, the effectiveness of SARS-CoV-2 diagnostics and therapeutics along with public health decision-making throughout the pandemic. However, as of April 2023, almost 20% of all SARS-CoV-2 sequences in GISAID were submitted by the UK alone, while only 2% came from Asian low- and middle-income countries (LMICs) (Afghanistan, Bangladesh, India, Iran, Nepal, Pakistan and Sri Lanka). Merely 0.1% were from the 22 countries of Africa, including Burundi, Comoros, Eritrea, Ethiopia, Madagascar, Malawi, Rwanda, Somalia and others. The Caribbeans contributed 0.06%, while LMIC in Latin America contributed 0.59%. These statistics underscore the importance of boosting SARS-CoV-2 sequence submissions from LMICs across Asia, Africa, the Caribbean and Latin America, to better understand virus diversity and shape effective pandemic strategies. Significantly, only a small portion of these sequences were produced, scrutinised and submitted by the LMICs. More often, patient samples from LMICs were sent to high-income countries (HICs) for sequencing, a stark testament to the unequal distribution of sequencing resources. According to GISAID data, a staggering 86.4%, 21.8% and 25.6% of samples from Afghanistan, Nepal and Pakistan, respectively, had to be shipped to HICs for sequencing. These figures highlight an urgent call to action for robust and sustained efforts in building genomics capacity. Despite the focus on pandemic-related research, the field of genomics still grapples with striking disparities—nearly 80% of human genomics studies centre around European ancestry, which represents less than 20% of the global population. This vast inequity signals a long, challenging road to achieving fair access to genomics. Moreover, to address this gap, studies on infections should strategically target populations bearing the heaviest disease burden, which has not always been the norm.^7 8^

Sequencing rapidly is not sufficient, especially in a pandemic setting. Modern public health systems are needed, equipped with robust surveillance capabilities to cater to the complexity of metadata collection during lockdowns, etc, as well as readiness to respond to pandemics, much like in the UK and Denmark. Countries may lack public health laboratories capable of handling samples of high infectious potential, or be overly centralised system and restricted to handle such samples (availability of biosafety facilities), which would cause sequencing and submission to be delayed.^7^ Limited resources and import restrictions can delay sequencing, while using outdated technology can lead to capacity issues and higher per-sample costs.^8^ LMICs often grapple with a lack of robust computational infrastructure and technical staff in academic institutions conducting in-depth public health genomics research. This is due to the nascent stage of the field of bioinformatics and software engineering in public health, coupled with insufficient professional development opportunities. Universities and public health institutions in such settings seldom create specialised roles for genomic epidemiologists and bioinformaticians.^9^ For sequencing data to effectively inform public health decisions, timely submission is crucial.

With over 220 million inhabitants, 65.6% aged over 15 years,^10^ Pakistan is a densely populated country significantly affected by the pandemic. Karachi, Pakistan’s largest city, saw its first COVID-19 case in February 2020.^11^ Since then, six COVID-19 waves have hit the nation, with the Delta and current Omicron variants causing significant surges. Only 87.26% of eligible adults are fully vaccinated, and 27.86% have received booster doses. As of April 2023, Pakistan has reported 1.58 million COVID-19 cases and 30 654 deaths.^12^ Pakistan’s genomic surveillance is insufficient, with only 6453 sequences on GISAID, equating to a mere 0.4% of total cases.^13^

Traditional North–South collaborations have involved HICs (the North) providing laboratory reagents to LMICs (the South), or LMICs sending biological samples to HICs for analysis. This one-sided exchange misses the essence of genuine, long-term partnership. Ideally, North-South collaborations should include knowledge and skill transfer, infrastructure development and empowerment of local institutions in LMICs. This way, these countries can gradually build their own capacity for research, rather than relying on resources and expertise from HICs.

We share our experience of an ideal collaboration, for instance with Chan Zuckerberg Biohub (CZB) and the Chan Zuckerberg Initiative (CZI), aimed at advancing in-house testing and analysis of sequencing data. In this context, we detail our journey of establishing genomic surveillance, sequencing infrastructure and the public dissemination of SARS-CoV-2 data under resource-restricted conditions. We also discussed the challenges we faced and how we resolved them. Our experiences and insights could serve as a blueprint for other LMICs exploring similar paths in the future.

## Building an in-house sequencing capacity

The Aga Khan University (AKU), a key private-sector educational institution in Pakistan, has significantly contributed to the local pandemic response through its clinical services, public outreach and extensive research activities. Prepandemic, the Department of Pediatrics and Child Health had ordered an Illumina iSeq100 for research, which arrived in March 2020. We received assistance from CZB/CZI in establishing our genomic infrastructure and training through webinars and Slack channel, reflecting true North-to-South collaboration. In late 2020, AKU chose to prioritise SARS-CoV-2 sequencing for the nation’s benefit.

### Sample collection and sequencing

Clinical nasopharyngeal samples that were positive for COVID-19 (with Ct values ≤25) were collected from the AKU Hospital (AKUH) clinical laboratories and sequenced at the Infectious Diseases Research Lab, Department of Pediatrics and Child Health, Medical College, AKU, Karachi, Pakistan, after informed consent. SARS-CoV-2 sequencing was performed using ARTIC-NEB (New England BioLabs) V.3 and V.4 protocols (dx.doi.org/10.17504/protocols.io.br77m9rn). Briefly, the Reverse Transcription Polymerase Chain Reaction (RT-PCR) based ARTIC protocol was used to obtain the amplicon from the extracted RNA, followed by the NEB FS DNA library preparation protocol to make the amplicon library. We successfully sequenced 705 SARS-CoV-2 on several batches using the Illumina iSeq100 and MiSeq platforms and collected the minimum required metadata (BioSample records by populating the mandatory fields^14^ under the Public Health Alliance for Genomic Epidemiology (PHA4GE) guidelines) on time.

#### Challenges, remedial actions and lessons learnt

##### Surveillance

The AKUH is one of the largest private tertiary care hospitals in the country with the largest network of laboratory collection points across the country. SARS-CoV-2 positive samples from the clinical laboratory formed the source of samples that fed into this surveillance. For positive samples from the inpatients, detailed contextual and clinical data could be extracted from the medical records. However, for outpatient samples, the metadata was mostly missing. The PHA4GE^15^ has developed an open-source SARS-CoV-2 contextual data specification package^14^ based on harmonisable, publicly available community standards. We adopted the same practice to consistently structure information as part of good data management practices, and for data sharing with trusted partners and/or public repositories. Clinical outcomes data were collected from the AKUH clinical patient database to correlate patients’ disease severity with variant type.

Standardisation, good data management practices and comprehensive data collection are vital in genomic epidemiology.

#### Procurement and maintenance of sequencing equipment

Procurement is a significant challenge in LMICs, including key equipment like sequencers. For most of the companies, the principal vendor is not located within the country, instead, they have distributors to do the business on their behalf where the duration of their distribution contracts varies. Therefore, we faced numerous challenges around price negotiation (received inflated prices), delivery timelines (5–6 months in our case for getting a sequencer), lack of on-site application and engineering support. We worked with our collaborators at CZB/CZI and the principal vendor (based in the USA) to overcome after-sales support and instrument troubleshooting issues. Since many distributors simply act as the primary source of technical support, it is essential to have complex technical support that is tailored to the requirements of the laboratory (availability of instrument spares and facility of troubleshooting within the country). There are, however, sporadic gaps in this support. The AKU’s engineering department and local vendors recently worked alongside the team from Illumina, UK, to remotely instal (calibrate and validate) NextSeq2000 in our laboratory to overcome this gap. Forming partnerships or consortiums, pooling resources and expertise to overcome the technical and logistical hurdles associated with such complex technology can help address these challenges in LMICs.

Obtaining sequencing reagents and consumables posed significant challenges. Distributors lacked local inventory, necessitating overseas imports, which were hampered by pandemic-related shortages and the recent Russia–Ukraine conflict. We are now collaborating with Illumina and their local distributor to secure a steady supply of sequencing reagents by building a local stockpile. This established relationship will facilitate other regional groups access to sequencing resources, thereby enhancing the region’s overall capacity.

Even before the pandemic, our laboratory faced issues with post-purchase support and services to install, maintain or calibrate systems to high standards. Our iSeq100 sequencer came with a 1-year warranty, but post-warranty, we could only replace the equipment under a special service contract. We did not experience any significant issues during the warranty, but it alerted us to the need for backup plans and adequate funds for equipment maintenance. We also secured a service contract with Illumina via our local distributor, stipulating that if the device fails, the days will not count towards the service contract and that the vendor would face a penalty if they were unable to address the malfunction. This arrangement meant that if our equipment failed for 3 months, our warranty was extended by an additional 4.5 months past its original termination date.

The key lesson from this experience is the critical importance of proactive planning and contractual protection for equipment maintenance and support in the context of genomic laboratories, particularly in regions with limited resources or infrastructure. Despite having a warranty, the foresight to secure a comprehensive service contract mitigated potential disruptions due to equipment failure and incentivised prompt vendor response. This highlights the necessity of strategic planning, adequate funding for equipment upkeep and strong contractual agreements to ensure continuous operation and service delivery in genomic research and public health scenarios.

#### Training

As recipients of the Gates Grand Challenges round 22 grant in May 2019, we had intended to visit CZB/CZI for training in sequencing and assay development for our metagenomics project, given our lack of previous in-house sequencing experience. However, the pandemic disrupted travel plans and we transitioned to online training, heavily relying on asynchronous communication through Slack due to time zone differences with the CZB/CZI team. Once travel restrictions eased, we were able to visit CZB/CZI in September 2022 for a 2-week intensive training on metagenomics, encompassing crucial aspects of laboratory work, analysis and epidemiology.

Remote learning and digital collaboration tools can be effectively leveraged when physical presence is not possible. However, our teams subsequent in-person visit for intensive training underscores the continued value of hands-on learning and direct interaction in building skills and knowledge in a complex field like metagenomics.

#### Library preparation

While preparing the library, we encountered problems related to adapter dimerisation, excessive DNA fragmentation and library quality control mandating adjustments to the sequencing protocol. These changes included increasing the number of magnetic bead cleanups to eliminate adapter dimers, reducing fragmentation time to prevent over-fragmentation of DNA and implementing a TapeStation system (a type of capillary electrophoresis system) to enhance library quality control (table 1).

**Table 1:** Potential solutions to overcome SARS-CoV-2 library preparation challenges

Challenge	Potential cause	Solution
Global supply chain issues (a complex network of manufacturing, processing and resource delivery)	Global shortage due to worldwide increase in sequencing kits/reagents for surveillance, plus pandemic-struck shipping causing overall supply chain issues.	We received a few supplies and reagents initially for the sequencing run from our international collaborators (CZB/CZI). Now, we are ordering even more in advance, or trying to order straight from the vendor instead of the distributor (less likely).
DNA input	Insufficient input of DNA (10 ng) was used per reaction.	Increased the input of DNA from 10 ng to 50 ng. Reduced barcoding PCR cycles from 14 to 12.
Very low yield of DNA even after concentrating them via beads	Beads were not resuspended properly, not adjusted to room temperature or accurate volume was not picked.	We completed further optimisation and quality control of the different SPRI bead ratios using ladders (untreated, 1.0×, 0.8×, 0.75×, 0.7×, 0.65× and 0.6×).
Adapter dimers issues on diagnostic PCR gel and TapeStation	Inappropriate fragmentation time of library preparation and/or SPRI beads clean up.	For the elimination of adapter dimers, we performed two SPRI clean-ups at 0.8× ratio after the barcoding PCR step. We also reduced the fragmentation time from 5 min to 3 min for library preparation.
Bands were not observed for diagnostic PCR	Adapters were not added, too low SPRI beads ratio, low concentration of samples on Qubit.	Adapters were added separately and not as part of a master mix, the corrected volume of SPRI beads was added during the clean-up.
Initial loading concentration	To avoid overloading, iSeq concentration range was set to 75 pM and 120 pM.	Set to optimal loading concentration of 100 pM of pooled library.
Problem in using BaseSpace sequence hub https://basespace.illumina.com	Low internet bandwidth while using BaseSpace sequence hub.	We used off-instrument implementation of Local Run Manager: https://support.illumina.com/sequencing/sequencing_software/local-run-manager/downloads.html
Quality control (QC) metrics of sequencing run	Due to internet bandwidth, we did not run QC online using BaseSpace.	QC metrics of sequencing run was done locally using Sequence Analysis Viewer (SAV) software: https://support.illumina.com/sequencing/sequencing_software/sequencing_analysis_viewer_sav/downloads.html

CZB, Chan Zuckerberg Biohub; CZI, Chan Zuckerberg Initiative; SPRI, Solid-phase reversible immobilization.

One must be prepared to conduct multiple experiments and adjust protocols as needed to ensure the quality of the results. In this case, interventions such as increasing the number of magnetic bead cleanups, adjusting the fragmentation time and integrating a TapeStation system for quality control were all crucial adjustments. This highlights the need for continuous learning, experimentation and adaptability in genomic research.

#### Lab space

The AKU core laboratory is a hive of activity, with devoted researchers, PhD students and PostDocs working in bacteriology, virology, parasitology and tissue banking sectors in the BSL-2 (Biosafety Level-2) and BSL-3 facilities. Despite these resources, operating in a core, multipurpose research laboratory designed to accommodate a wide array of life sciences research presented a significant challenge, particularly due to the lack of dedicated areas for nucleic acid extraction, PCR clean space, post-PCR space, sequencing space and so on. Initially, our collaborators recommended a BSL-3 space, prompting us to create segregated areas within the BSL-3 facility for RNA extraction, minimising contamination risks between samples and library preparations.

Specific processes like nucleic acid extraction, PCR and sequencing require isolated spaces to prevent cross-contamination and ensure data integrity. Adaptive solutions, like creating separated areas within existing facilities when setting up new, dedicated spaces is not feasible.

With the work we have done so far, we have been funded to establish a state-of-the-art molecular and sequencing facility within the new Women and Child Health Building, AKU Karachi.

#### Bioinformatics analysis

On 16 April 2021, we received a grant from PHA4GE to implement standardised bioinformatics practices, pipelines and data structures in SARS-CoV-2 sequencing laboratories in Pakistan. Sequences were analysed to obtain SARS-CoV-2 consensus genome assemblies using the in-house deployment of Chan Zuckerberg ID (CZ ID) consensus genome mini-Workflow Descriptive Language (WDL)-based pipeline on the AKU server (~8–9 mins on each sample). We uploaded our experiences of installation of bioinformatics protocols at https://waqasnayab.gitbook.io/pipeline/master/implementation-of-cz-id-mini-wdl-based-sars-cov-2-consensus-genome-workflow-pipeline-at-aku.

The shortage of bioinformatics expertise in Pakistan is another obstacle. As part of another Bill & Melinda Gates Foundatiuon (BMGF)-funded project, we had been building the bioinformatics capacity for the last couple of years preceding the pandemic. This came to good use during the pandemic. During the implementation of the CZ ID mini-WDL-based command line interface (CLI) pipeline, we faced several roadblocks that were discussed and resolved at PHA4GE #pakistan-aga-khan slack channel (https://pha4ge-subgrant.slack.com/archives/C029C32JGKX) with PHA4GE Bioinformatics Pipelines and Vizualisation working group. Table 2 described the challenges and risk-mitigated strategies specific to the deployment of the CZ ID pipeline. As viral genomes mutate, new variants are continuously emerging. To integrate new information from bioinformatics databases along with their related analysis, pipelines need to be continually evolving. For the most up-to-date analysis pipeline, the locally installed CZ ID pipeline is updated after every 2–3 weeks.

**Table 2:** Roadblocks and strategies to encounter that appeared during the deployment of Chan Zuckerburg ID SARS-CoV-2 consensus genome pipeline on AKUs’ existing infrastructure

Roadblock	Description	Risk mitigation strategy	Links
Docker downloading issue	While downloading essential files (reference, primer, kraken and VADR) using docker from s3 amazon server, encounter an issue in downloading reference genome.	The issue was specific to Linux environment, hg38.fa.gz was downloaded on windows environment and then transferred to Linux workspace.	https://github.com/chanzuckerberg/idseq-workflows/issues/177
Export mini-WDL cache folder	The essential files required as input in CZ ID pipeline are downloaded and stored in ‘mini-wdl_download_cache’ folder. This was a one-time download which took ~4 hours to complete. But in our case, those files were downloading and over-riding ‘mini-wdl_download_cache’ folder repeatedly at every run.	A query regarding this issue was posted on CZ ID GitHub page. The issue was solved by incorporating additional parameters to main mini-WDL run command.	https://github.com/chanzuckerberg/idseq-workflows/issues/179
Creation of extraneous control characters	A six extraneous control characters folder was created that aborted the pipeline in the middle.	A query was posted on CZ ID GitHub page. The issue was solved by defining the path for storing cache files. The developers then updated their GitHub page accordingly.	https://github.com/chanzuckerberg/idseq-workflows/issues/142
Updating CZ ID consensus genome workflow	The developers made changes in the main code by updating iVar consensus threshold value from 0.9 to 0.75. (75% base frequency is required for a call, otherwise resulted in mixed sites).	CZ ID pipeline was updated locally, and new changes were incorporated.	–
CZ ID multi sample run	Processing all samples at a time and obtaining combined consensus genome file with combined vcf file is currently not possible through mini-WDL consensus genome workflow.	In-house bash script was written to process multiple samples at a time using a single command.	–
Timestamp directory	Mini-WDL pipeline creates a timestamp consensus genome directory for every sample in which all intermediate and final output folders are stored. This was a challenge since we were running the sample in batches and timestamp directory was creating difficulty in matching output files with specific sample ID.	-d parameter in mini-WDL pipeline with trailing slash and dot can be used to specify and create the folder having name of choice. In this way, we renamed the directory as sample id instead of timestamp.	https://github.com/chanzuckerberg/czid-workflows/issues/33

AKU, Aga Khan University; CZ ID, Chan Zuckerburg ID ; VADR, Variant Analysis of DNA Repair; WDL, Workflow Descriptive Language.

Adapting standardised practices for implementation of a bioinformatics pipeline is essential. Accessing open resources, and in turn being open to resource sharing of experiences and protocols publicly can help countries seeking to implement similar pipelines and infrastructure. Prior investment in building this capacity proved invaluable during the pandemic, underlining the need for long-term capacity-building efforts, even before crises occur. The role of global collaborative networks and digital tools is critical to regularly updating locally installed pipelines ensuring accurate and current analysis.

#### Data sharing

Promptly uploading the data to GISAID for proactive pandemic response is crucial. While uploading sequences to GISAID, the true value lies in timely submission allowing for real-time analysis that can inform public health interventions. Countries with robust public health systems may have an advantage in efficiently managing sample collection and sequencing processes. We had mixed success in rapid sharing of data. As of 14 April 2023, the median lag time between sample collection and upload to GISAID was 36 days.

Consensus genomes are widely deposited in open-access databases to use for public surveillance purposes. On the other hand, raw data is critical for comparing methods and assessing reproducibility, as well as identifying minor variants. Most SARS-CoV-2 sequences (consensus genomes) have only been deposited in GISAID, with a proportion of submitters also depositing matching raw read data in the International Nucleotide Sequence Database Collaboration that includes (1) National Center for Biotechnology Information (NCBI), (2) European Molecular Biology Laboratory–European Bioinformatics Institute and (3) DNA Data Bank of Japan. Linkage of contextual data to consensus sequences as well as raw data in public repositories is vital. At present, we successfully uploaded high-quality SARS-CoV-2 viral sequences and related metadata to open-source sequencing databases, that is, GISAID, GenBank and European Bioinformatics Institute’s COVID-19 portal. We are the first laboratory from Pakistan that submitted sequencing data in the form of raw paired-end fastq files for researchers’ community benchmarking of evolving bioinformatics tools and algorithms (BioProject accession ID: PRJNA764553).

A major challenge that was encountered with the submission of SARS-CoV-2 consensus genome sequences on GISAID was the presence of frameshift mutations and mixed sites observed in some samples. The quality of viral FASTA sequences was evaluated using Nextclade (https://clades.nextstrain.org/), sequences with Ns more than 3000 were discarded altogether, however, sequences having mixed sites and frameshift issues were corrected and proceeded for submission. To improve the genome quality, the following in-house methods were used:

- Frameshift mutations were corrected by an in-house analytical method. As the sample sequence was aligned with the reference genome, the site of frameshift was visualised. The pipeline-induced indels were corrected manually in the FASTA file.
- Mixed sites in each sample were identified by aligning them with the reference genome. The location of the identified mixed site was then manually checked in the BAM file using IGV V.2.10.^16^ If nucleotide frequency at that position crossed a certain acceptable threshold (0.75), it was corrected in the consensus genome FASTA file.

#### Data dissemination

**Figure 1:**
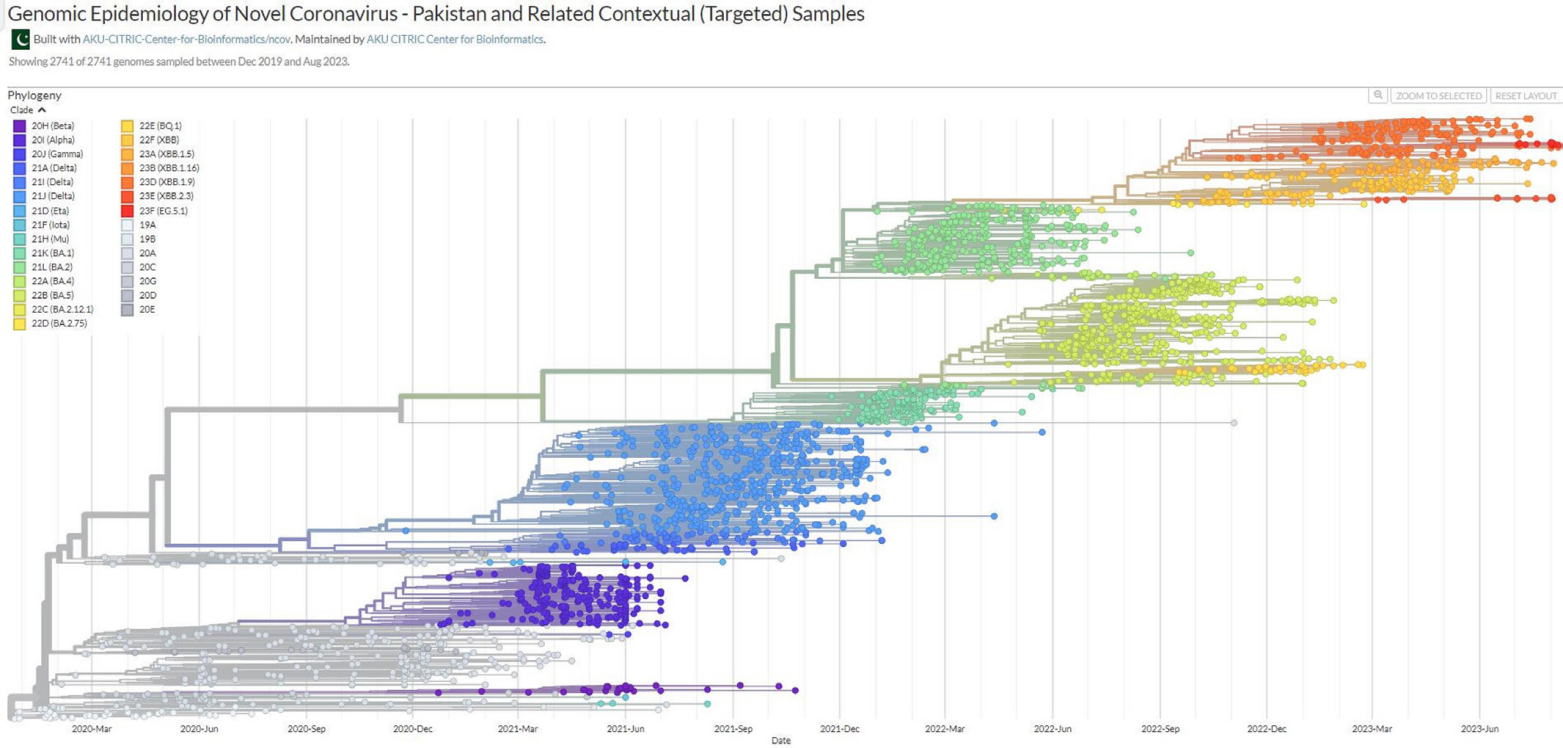
Genomic epidemiology of SARS-CoV-2 - Pakistan and related contextual samples at Nextstrain.

SARS-CoV-2 has swept globally, and Pakistan is no exception. Where the virus was introduced, and how it was transmitted in the early stage of the epidemic in Pakistan is largely unknown. Genomic epidemiology is a powerful tool to answer these questions. However, owing to the scarcity of testing and lack of sequencing of samples in an LMIC setting, Pakistan was poorly represented on the Nextstrain global build. Our research group under the umbrella of CITRIC Center for Bioinformatics and Computational Biology, Karachi campus, decided to construct a Pakistan-specific build to get a complete map of SARS-CoV-2 genomic epidemiology in Pakistan. For this, a snakemake-based Nextstrain’s CLI pipeline was installed on AKU’s server. We successfully deployed a phylosurveillance map of Pakistan (Pakistan-focused Subsampling; Non-contextual/Non-targeted) as a Nextstrain build (https://nextstrain.org/community/AKU-CITRIC-Center-for-Bioinformatics/ncov/Pakistan) in October 2021 (figure 1). This endeavour is the country’s very first contribution to the field of Genomic Epidemiology. In November 2021, we also added Pakistan and Related Contextual (Targeted) Samples build (https://nextstrain.org/community/AKU-CITRIC-Center-for-Bioinformatics/ncov/Pakistan-Contextual). Both builds are updated after every 2–3 weeks (depending on the number of sequences submitted from Pakistan, https://github.com/AKU-CITRIC-Center-for-Bioinformatics/ncov/releases).

The implementation of Nextstrain build in a low-resource setting, particularly in LMICs, presented several challenges. While executing the pipeline to construct the Pakistan-specific tree, we encountered various errors. One of the significant hurdles was the limited availability of local personnel with bioinformatics expertise. This scarcity of skilled personnel is a common issue in LMICs where specialised training programmes may be lacking, that is, phylogenetics analysis. Moreover, infrastructure limitations posed additional obstacles, such as, the requirement of a suitable server (72 cores machine took ~3.5 hours for Pak-build) with a reliable internet connection (weekly update to latest versions of pipelines). These infrastructure elements are crucial for handling and processing Nextstrain analysis. Overcoming these challenges necessitated seeking external support and exploring avenues for training programmes to enhance local bioinformatics capabilities. In our case, a meeting was arranged with Nextstrain’s developer, who provided valuable guidance in resolving the encountered issues:

- GISAID has a few sequences submitted from Pakistan which created hurdles in building the tree using the default main script (builds.yml). The yml script was edited and customised to make it executable on Pakistani sequences data.
- Deployment of Pakistan build on the Nextstrain webpage was also a challenge. A pull request was made on Nextstrain’s GitHub page and was fetched by a member of the developer team to merge and deploy the tree on Nextstrain (https://github.com/nextstrain/nextstrain.org/pull/416).

Implementing a phylosurveillance map in Pakistan was an important local contribution to the field of genomic epidemiology, demonstrating that even in resource-limited settings, significant contributions to global understanding can be made. Using open-source tools like Nextstrain’s CLI pipeline can help resource-limited settings to implement genomic surveillance and contribute to global understanding of the virus. Empowerment through self-initiative can be an enabler.

## The way forward

Since the initiation of this work, the team has started SARS-CoV-2 sequencing for wastewater-based epidemiology, another area of a public health laboratory research and implementation, that needs immediate attention. We have also embarked on other microbial sequencing including microbiome, nasopharyngeal swabs for *Pneumococcal*, *Klebsiella* and other enteric pathogens.

In establishing these processes, sustainability has been a major challenge with funding being the biggest concern. Advocacy is a pivotal component; promoting understanding and importance of genomics in policy discussions can garner more sustained funding and support. Diversification is also key where the reliance must go beyond external grants. Funding from public–private partnerships, local government bodies or collaborations with international universities or research institutions can play a huge role. Concurrently, we need to ensure our research is translated into tangible applications in various sectors, such as public health, agriculture and environmental science, to attract significant funding. Transforming the sequencing centre into a service provider could generate income that supports the continuation of our operations.

It is crucial to invest in the training and development of local scientists and technicians to cultivate domestic expertise and reduce reliance on external resources. A proactive approach is required to plan for maintenance and updates of sequencing technology to prevent expensive and disruptive system failures or replacements active engagement with the global genomics community is essential to do this as it offers avenues for resource exchange, learning opportunities and potential partnerships.^17^

The government must employ critical steps to establish a centralised procurement system for wet-lab reagents and NGS platform components, such as those from Illumina. Collaborations with initiative teams, like CZB/CZI, can add significant value.

Going forward, we aim to apply the lessons from the COVID-19 pandemic to future strategies against emerging and evolving pathogens. The value of genomics in controlling disease outbreaks has been highlighted, and our goal is to establish robust genomic response programmes for future pandemics and disease outbreaks. By leveraging our updated equipment and high-performance computing infrastructure, we aim to develop tools and pipelines for diverse pathogen genomic analyses, enabling us to face future challenges more effectively.

## Data Availability

Data are available in a public, open access repository. Data sharing not applicable as no data sets generated and/or analysed for this study. The sequencing data is available under BioProject accession ID: PRJNA764553

## References

[1] Franke KR, Isett R, Robbins A, et al. Genomic surveillance of SARS-Cov-2 in the state of delaware reveals tremendous Genomic diversity. PLoS One 2022;17:e0262573. 10.1371/journal.pone.0262573 35045124 PMC8769358

[2] Wu F, Zhao S, Yu B, et al. Author correction: a new coronavirus associated with human respiratory disease in China. Nature 2020;580:E7. 10.1038/s41586-020-2202-3 32296181 PMC7608129

[3] Okada P, Buathong R, Phuygun S, et al. Early transmission patterns of coronavirus disease 2019 (COVID-19) in travellers from Wuhan to Thailand. Euro Surveill 2020;25. 10.2807/1560-7917.ES.2020.25.8.2000097 PMC7055038 32127124

[4] Lo SW, Jamrozy D. Author correction: genomics and epidemiological surveillance. Nat Rev Microbiol 2020;18:539. 10.1038/s41579-020-0428-6 PMC7608226 32724190

[5] GISAID. Available: https://www.gisaid.org/

[6] Oude Munnink BB, Worp N, Nieuwenhuijse DF, et al. Author correction: the next phase of SARS-Cov-2 surveillance: real-time molecular epidemiology. Nat Med 2021;27:2048. 10.1038/s41591-021-01567-4 PMC8491748 34611328

[7] Brito AF, Semenova E, Dudas G, et al. Global disparities in SARS-Cov-2 genomic surveillance. medRxiv 2021. 10.1101/2021.08.21.21262393 PMC9667854 36385137

[8] Kalia K, Saberwal G, Sharma G. The lag in SARS-Cov-2 genome submissions to GISAID. Nat Biotechnol 2021;39:1058–60. 10.1038/s41587-021-01040-0 34376850

[9] Black A, MacCannell DR, Sibley TR, et al. Ten recommendations for supporting open pathogen genomic analysis in public health. Nat Med 2020;26:832–41. 10.1038/s41591-020-0935-z 32528156 PMC7363500

[10] Pakistan Bureau of Statistics. Available: https://www.pbs.gov.pk/

[11] Abid K, Bari YA, Younas M, et al. Progress of COVID-19 epidemic in Pakistan. Asia Pac J Public Health 2020;32:154–6. 10.1177/1010539520927259 32429679 PMC7240311

[12] COVID-19 health advisory platform by Ministry of national health services regulations and coordination. Available: https://covid.gov.pk/

[13] Vavrek D, Speroni L, Curnow KJ, et al. Genomic surveillance at scale is required to detect newly emerging strains at an early Timepoint. medRxiv [Preprint] 2021. 10.1101/2021.01.12.21249613

[14] Griffiths EJ, Timme RE, Mendes CI, et al. Future-Proofing and maximizing the utility of metadata: the Pha4Ge SARS-Cov-2 contextual data specification package. Gigascience 2022;11:giac003. 10.1093/gigascience/giac003 35169842 PMC8847733

[15] PhA4GE. Genomic epidemiology. 2021. Available: https://pha4ge.org/ [Accessed 8 Jun 2022].

[16] Robinson JT, Thorvaldsdóttir H, Wenger AM, et al. Variant review with the integrative genomics viewer. Cancer Res 2017;77:e31–4. 10.1158/0008-5472.CAN-17-0337 29092934 PMC5678989

[17] Sahadeo NSD, Nicholls S, Moreira FRR, et al. Implementation of genomic surveillance of SARS-Cov-2 in the caribbean: lessons learned for sustainability in resource-limited settings. PLOS Glob Public Health 2023;3:e0001455. 10.1371/journal.pgph.0001455 36963002 PMC10022082

